# Antibody avidity, persistence, and response to antigen recall: comparison of vaccine adjuvants

**DOI:** 10.1038/s41541-021-00337-0

**Published:** 2021-05-21

**Authors:** Sonia Budroni, Francesca Buricchi, Andrea Cavallone, Patricia Bourguignon, Magalie Caubet, Vincent Dewar, Ugo D’Oro, Oretta Finco, Nathalie Garçon, Mohamed El Idrissi, Michel Janssens, Geert Leroux-Roels, Arnaud Marchant, Tino Schwarz, Pierre Van Damme, Gianfranco Volpini, Robbert van der Most, Arnaud M. Didierlaurent, Wivine Burny

**Affiliations:** 1 GSK, Siena, Italy; 2 GSK, Rixensart, Belgium; 3 Bioaster Technology Research Institute, Lyon, France; 4 Center for Vaccinology, Ghent University, Ghent, Belgium; 5 Institute for Medical Immunology, Université libre de Bruxelles, Brussels, Belgium; 6 Institute of Laboratory Medicine and Vaccination Center, Klinikum Wuerzburg Mitte, Standort Juliusspital, Academic Teaching Hospital of the University of Wuerzburg, Wuerzburg, Germany; 7 Center for the Evaluation of Vaccination, Vaccine and Infectious Disease Institute, University of Antwerp, Antwerp, Belgium; 8 Present Address: GSK, Wavre, Belgium; 9 Center of Vaccinology, University of Geneva, Geneva, Switzerland

**Keywords:** Medical research, Translational research, Immunology, Adaptive immunity, Vaccines

## Abstract

Differences in innate immune ‘imprinting’ between vaccine adjuvants may mediate dissimilar effects on the quantity/quality of persisting adaptive responses. We compared antibody avidity maturation, antibody/memory B cell/CD4^+^ T cell response durability, and recall responses to non-adjuvanted fractional-dose antigen administered 1-year post-immunization (Day [D]360), between hepatitis B vaccines containing Adjuvant System (AS)01_B_, AS01_E_, AS03, AS04, or Alum (NCT00805389). Both the antibody and B cell levels ranked similarly (AS01_B/E_/AS03 > AS04 > Alum) at peak response, at D360, and following their increases post-antigen recall (D390). Proportions of high-avidity antibodies increased post-dose 2 across all groups and persisted at D360, but avidity maturation appeared to be more strongly promoted by AS vs. Alum. Post-antigen recall, frequencies of subjects with high-avidity antibodies increased only markedly in the AS groups. Among the AS, total antibody responses were lowest for AS04. However, proportions of high-avidity antibodies were similar between groups, suggesting that MPL in AS04 contributes to avidity maturation. Specific combinations of immunoenhancers in the AS, regardless of their individual nature, increase antibody persistence and avidity maturation.

## Introduction

Protection against many infectious diseases is mediated by a functional, persistent antibody response, which is thus a critical immune correlate for many licensed human vaccines. The durability of vaccine-acquired antibody responses varies greatly between antigens^1^. Antibodies induced by viral infections, or by vaccination with live-attenuated viruses, can persist for decades^2^. However, most vaccines based on protein antigens require repeated immunizations to generate immunological memory, and to maintain antibody responses above protective levels^2^. The level of antigen–antibody binding avidity, a qualitative response index, can also correlate with protection. This has been demonstrated for the RTS, S malaria vaccine, amongst others, and for several monoclonal antibodies (mAb) treatments^3,4^. Reversely, low-avidity antibodies have been associated with antibody-mediated disease enhancement following a certain respiratory syncytial virus, dengue, or pandemic influenza vaccinations^5^. Moreover, inadequate levels of avidity maturation (the latter defined as the increase of avidity over time) can heighten susceptibility to viral infection, as seen for mumps vaccines^6^. Thus, both quantitative and qualitative yardsticks can determine vaccine efficacy.

Vaccine adjuvants, such as oil-in-water emulsions or Toll-like receptor (TLR) agonists, are linked to both of these aspects of the antibody response. By enhancing innate immunity, including responses of stimulated antigen-loaded antigen-presenting cells (APCs), they promote activation of naive B cells and CD4^+^ T cells^7^. Potentially mediated through increased T cell receptor (TCR) avidity, and via enhanced interactions with dendritic cells and B cells, CD4^+^ T cells can differentiate into T follicular helper (T_FH_) cells. The latter cells are thought to drive B cell differentiation, most likely at the plasmablast level^8^. In turn, differences in plasmablasts translate into qualitative differences in the B cells responsible for long-term antibody maintenance, increased avidity, and expanded repertoires for immediate antibody production^9–11^. Enhancement of innate signaling can be obtained via a specific adjuvant combination (a GSK proprietary ‘Adjuvant System’, or AS). Examples are AS01_B_ (TLR4 ligand 3-*O*-desacyl-4′-monophosphoryl lipid A [MPL] and QS-21 formulated into cholesterol-containing liposomes), AS01_E_ (half-dose AS01_B_ with respect to MPL and QS-21 quantities), the α-tocopherol–containing oil-in-water emulsion AS03, and AS04 (aluminum salt [Alum] and MPL)^12–15^. All four AS are currently used in licensed and candidate vaccines^7^.

Antibody avidity (as well as titers) can also be significantly enhanced by repeated immunization, particularly when the vaccine contains an effective adjuvant^3,10,16,17^. To counter vaccine shortages and stretch vaccine supplies, the feasibility of booster immunizations with reduced antigen and adjuvant doses has been evaluated^18,19^. However, the effects of the antigen or adjuvant dose used in such boosters, on their effectiveness, remain under-researched.

Using adjuvanted vaccines based on hepatitis B surface antigen (HBsAg) in healthy hepatitis B virus (HBV)-naive participants, we previously reported head-to-head comparisons of the gene expression and innate and adaptive immune responses between the four AS^12,20,21^. The licensed Alum-adjuvanted HBsAg vaccine was thereby used as a comparator. We found a general ranking of adaptive and innate response levels between the five adjuvants (AS01_B_ ≥ AS01_E_ > AS03 > AS04 > Alum). In addition, we detected a core innate gene signature, which emerged after the second vaccination in AS01_B/E_ and AS03 recipients. This signature was characterized by positive regulation of genes associated with innate-cell and interferon (IFN)-related responses, and by negative regulation of NK cell-associated genes in the blood. The presence of this signature, and the early CRP, IL-6, and IFN-related serum responses, correlated with the magnitude of HBsAg-specific antibody responses.

In the present exploratory study, we evaluated how these adjuvants compare with respect to the durability of antibody concentrations and memory B cell and CD40L^+^ CD4^+^ T cell responses in blood, up to one-year post-vaccination. We also compared the adjuvants with respect to their capacities to elicit functional immune memory, by evaluating the response upon non-adjuvanted antigen recall. To this end, we administered the third vaccination with HBsAg alone, containing 1/4th of the standard (20 µg) vaccine antigen dose, at one-year post-dose 1. We hypothesized that the different levels of innate stimulation by the adjuvanted prime-boost vaccinations result in differences in any elicited T_FH_ subsets. These differences will then translate into variable levels of memory B cell ‘imprinting’, which in turn lead to quantitative and qualitative differences in persisting and recall responses. Indeed, long-lived plasma cells (PCs) elicited by the first two vaccine doses and residing amongst others in the bone marrow (BM), have been shown to provide long-term antibody maintenance^22^. However, it is expected that memory B cells, differentiated into short-lived PCs, would be responsible for the immediate antibody response post-antigen recall. Of note, levels of a key enzyme in B cell somatic hypermutation and antibody avidity maturation were shown to be increased following vaccination^23^. Thus, as antigen exposure modulates the B cell response, any antibody quality measurements after the antigen recall are likely to reflect qualitative changes in memory B cells.

Assessment of vaccine-elicited immunity was first performed for quantitative response levels, and then qualitatively by characterizing the kinetics of avidity maturation of polyclonal antibody populations in the sera. Avidity was quantified using an innovative method^24,25^ (submitted manuscript) to model the antibody-antigen reaction profiles obtained by a microfluidic ligand-binding assay.

The results contribute to our current knowledge of the effect of adjuvants on antibody quality and support the selection of adjuvants and optimized immunization regimens in clinical vaccine development.

## Results

### Durability and proliferation capacity of memory B/T cells are independent of the adjuvant type

We first compared, between adjuvant groups, the durability of immune memory up to one-year post-dose 1 (Day [D]360) in the ‘Booster’ cohort (*N* = 265). HBsAg-specific antibodies, memory B cells, and CD40L^+^ CD4^+^ T cells were assessed. Subjects received two doses of HBsAg vaccine adjuvanted with AS01_B_, AS01_E_, AS03, AS04, or Alum, at D0 and D30^21^. Then, to investigate memory functionality, we contrasted the recall responses to low-dose non-adjuvanted HBsAg administered at D360.

**Fig. 1:**
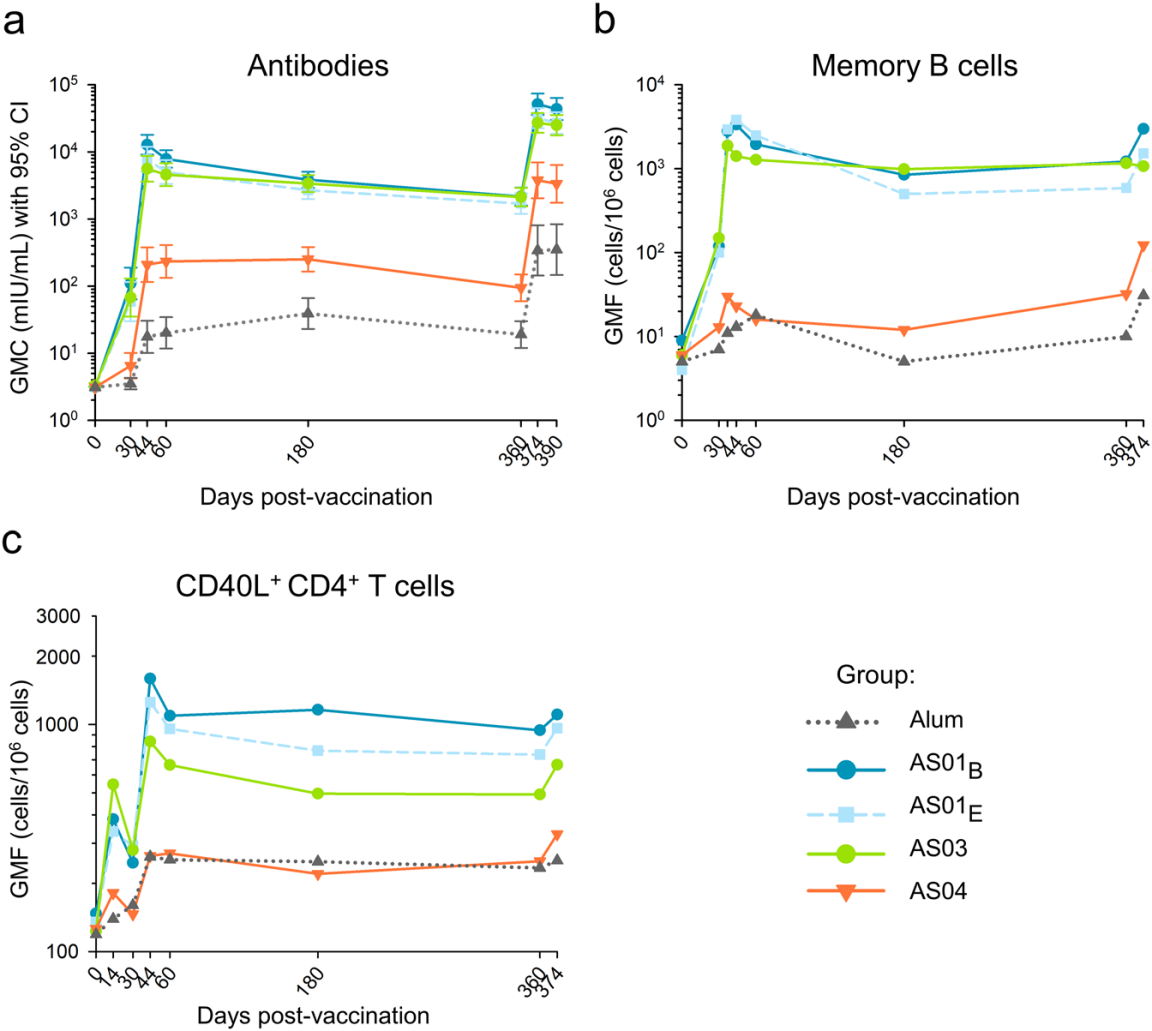
Persistence and post-antigen recall responses of HBsAg-specific antibodies, memory B cells, and CD4^+^ T cells in the Booster Cohort. Subjects of the per-protocol Booster Cohort (*N* = 265) received two doses of hepatitis B surface antigen (HBsAg) vaccine adjuvanted with AS01_B_, AS01_E_, AS03, AS04, or Alum at Day (D)0 and D30. They also received revaccination with non-adjuvanted HBsAg, using 1/4th of the adjuvanted antigen dose, at D360. Lines and symbols are color-coded according to the adjuvant groups presented in the key. Presented are the kinetics of geometric mean concentrations (GMC) of anti-HBsAg antibodies with 95% confidence intervals (CI; **a**), and the kinetics of geometric mean frequencies (GMF) of HBsAg-specific IgG-secreting memory B cells (**b**) or CD40L^+^ CD4^+^ T cells (**c**).

**Fig. 2:**
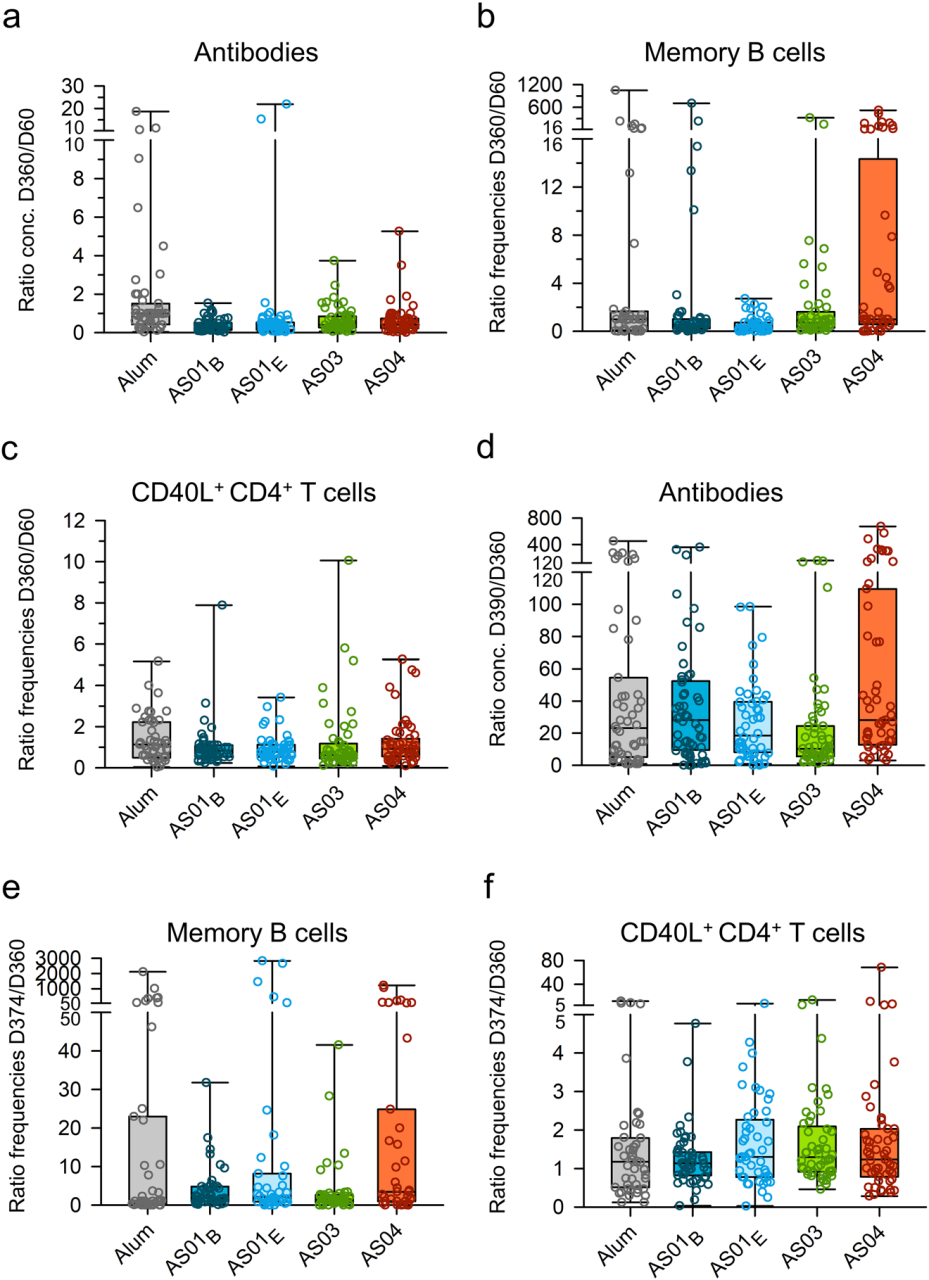
Persistence and post-antigen recall responses of HBsAg-specific antibodies, memory B cells, and CD4^+^ T cells in the Booster Cohort. Subjects of the per-protocol Booster Cohort (*N* = 265) received two doses of hepatitis B surface antigen (HBsAg) vaccine adjuvanted with AS01_B_, AS01_E_, AS03, AS04 or Alum at Day (D)0 and D30, and revaccination with non-adjuvanted HBsAg at D360. Ratios of antibody concentrations and B cell or T cell frequencies are presented as D360 over D60 (**a**–**c**), and as D390 or D374 over D360, i.e., post/pre-antigen recall (**d**–**f**). Boxplots represent medians, first and third quartiles, minima, and maxima. Each symbol represents an individual subject.

Based on geometric means of concentrations/frequencies (GMCs/GMFs) at D360, all groups displayed persisting HBsAg-specific antibody, memory B cell, and CD4^+^ T cell responses (Fig. 1a–c). For HBsAg/Alum and HBsAg/AS01_B_, this was consistent with data for other populations^26–28^. For all three immune parameters, we observed at D360 only slight changes from the peak levels measured at D60, irrespective of the adjuvant (Fig. 2a–c). With respect to memory B cell responses, the larger third quartiles (Q3) in the AS04 group suggested greater interindividual variability for this AS than for the other AS. Similar observations were made for the innate and adaptive responses following the second dose of HBsAg/AS04^20,21^. Since the proportional changes over time were largely similar between groups, the quantitative differences in peak responses persisted at D360 (AS01_B/E_/AS03 > AS04 > Alum [antibodies]; AS01_B/E_ ≥ AS03 > AS04/Alum [B/T cells]). Together with the minor changes in memory B cell and antibody GMFs/GMCs from D60 to D360, the data suggest ongoing antibody production. Possibly this aligns with the 150-day half-life of HBsAg antibodies in healthy adults^29^. In addition, the data suggest that functional immune memory was induced and maintained. The latter was confirmed by the robust anamnestic antibody responses seen in all groups two weeks after revaccination with HBsAg alone (D374), which persisted at D390. Since all groups had comparable proportional changes in GMCs from D360 to D390, they ranked similarly at D390 as they did at D60 or D360. Relative to antibodies, changes in memory B cell and CD4^+^ T cell GMFs were smaller, and D374 levels did not substantially exceed respective peak levels in all groups except the AS04 group. In the latter group, the revaccination induced a strong boost of, particularly memory B cell responses. As for GMCs/GMFs, median fold-changes in pre/post-recall responses were substantially larger for antibodies than for B and T cells (Fig. 2d–f). The interindividual variability in the D374/D360 ratios of antibody and memory B cell responses was greater for AS04 than for the other AS. For the memory B cell responses, this aligned with the D360/D60 ratios. This suggests that the inter-individual differences between the AS04 recipients that were present at D360 were fine-tuned and amplified after the revaccination. Along with the above-described data (Figs. 1, 2a–c), this hints at a different behavior of AS04.

Antibody secretion shortly after antigen recall is thought to depend on restimulated memory B cells that become short-lived PCs^22^. In addition to these direct responses, D390 antibody concentrations will also depend on quantities of pre-existing (D360) antibodies, thought to be exclusively maintained by longlived BM PCs operating independently of memory B cells or antigen stimulation. Our data hint at a low-to-moderate association between D360 memory B cell levels, and increases in serum antibody concentrations upon revaccination (∆D390-D360) (Supplementary Fig. 1). These associations appeared to be more prominent for AS01 and AS03 than for AS04 or Alum. Indeed, several recipients of Alum (*N* = 24) or AS04 (*N* = 16) displayed increased antibody responses without memory B cell responses. Similar trends were seen in other populations, both for responses to HBsAg/Alum and, though infrequently, for responses to AS04-adjuvanted human papillomavirus (HPV) vaccine^30–32^. The differences between groups may be consistent with our hypothesis that, due to dissimilar innate signals provided to CD4^+^ T cells by the adjuvanted vaccines, the different levels of T cell help received by memory B cells result in short-lived PCs with diverging qualities, for example in terms of affinity maturation^11,23^. However, whether such differences translate into dissimilar antibody-production capacities by these cells is unknown. In addition, the data may be obfuscated by the variability of the B cell enzyme-linked immunosorbent spot (ELISPOT) assay^33^.

Overall, the differences between the adjuvants displayed in the magnitudes of humoral and cellular responses at the peak were maintained with respect to the long-term and recall responses (Fig. 1). The adjuvants may however differ in their abilities to promote functional B cell memory responses. To that aim, we next compared the adjuvants’ effects on antibody avidity, using a cohort subset (*N* = 99). Relative to the full cohort, this subset exhibited comparable kinetic patterns in humoral and cellular responses (Supplementary Figs. 2 and 3). However, as compared to the full dataset, variability in D374/D360 fold-changes in memory B cell frequencies appeared higher for Alum, and lower for AS04 (Supplementary Fig. 3e).

### Antibody avidity analysis

**Fig. 3:**
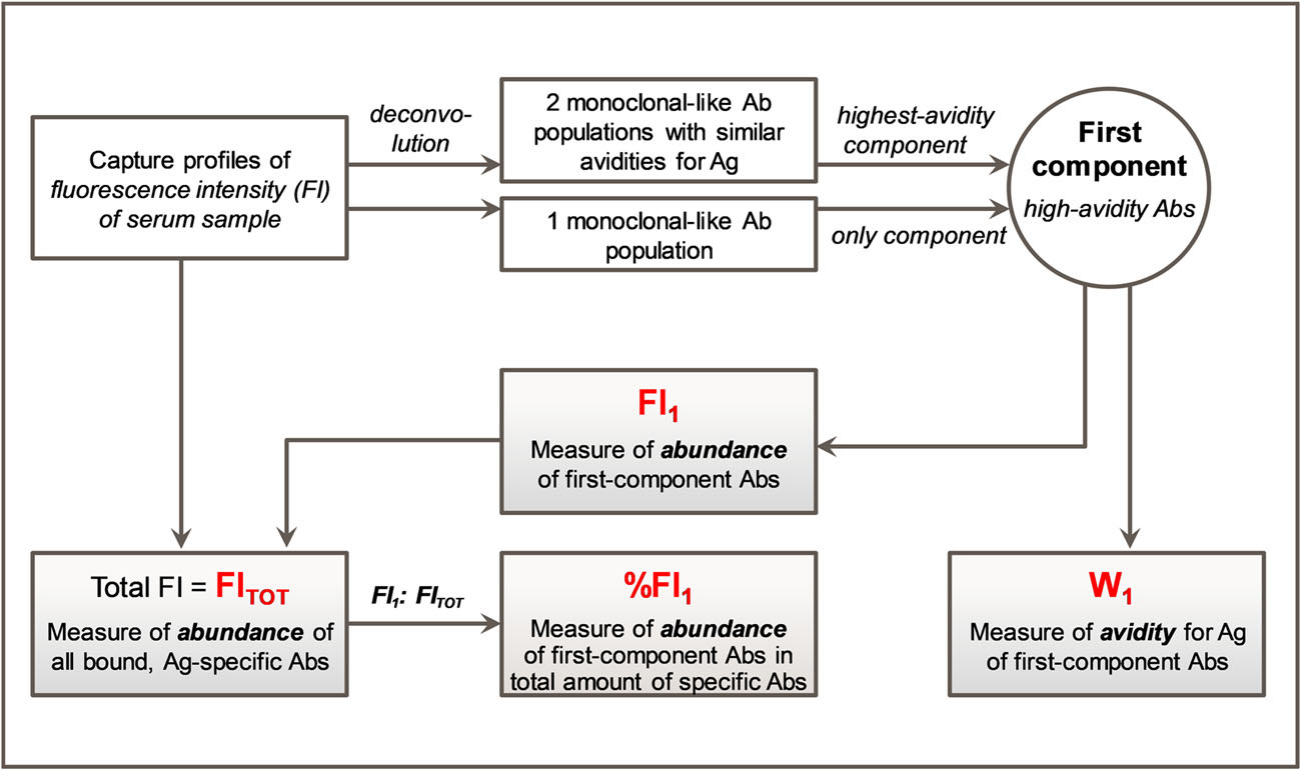
Summary of the data analysis strategy and main parameters. Figure summarizes the data analysis pipeline applied to characterize the avidity of polyclonal antibody (Ab) populations in sera obtained after vaccination (see Suppl. Fig. 5 and “Methods” section for details). Fluorescence intensity (FI) data representing the antigen (Ag)-Ab binding (antibody ‘capture profiles’) were obtained by microfluidic ligand-binding immunoassay. The area under the capture profile represented the total amount of HBsAg-specific antibodies in the sample (FI_TOT_). Modeling of the capture profiles using a deconvolution algorithm-generated one or two monoclonal-like Ab populations with similar avidities (‘components’). Abs in the highest-avidity (‘first’) component is characterized by %FI_1_ (FI contribution from these Abs [FI_1_] relative to the FI_TOT_), and the avidity score W_1_ representing the binding strength of these Abs. Parameters indicated in red font were used to characterize the abundance and avidity of the sera. The cutoff for high-avidity Abs was defined as W_1_ < 5, corresponding to approximately K_D_ < 10^−10^ M.

Antibody avidity for HBsAg of the sera was quantified using an adaptation of a high-throughput affinity assay initially developed for mAbs and Fabs^24,25^. Repeated runs of a serum sample by microfluidic ligand-binding assay^34^ resulted in a set of fluorescence intensity (FI) distributions (‘antibody capture profiles’), from which an averaged capture profile was computed. For each sample, the total FI (FI_TOT_) value was estimated from the area under the averaged capture profile. A plot of the FI_TOT_ values against the corresponding CLIA antibody concentrations demonstrated a correlation between the two parameters (Supplementary Fig. 4). We then subjected the averaged profiles to a mathematical modeling and analysis strategy (summarized in Fig. 3, see Supplementary Fig. 5 and “Methods” section for details). Deconvolution of the averaged capture profiles allowed separating a sample’s antibody population into a higher-avidity (first) and a medium-to-low-avidity (second) component. The FI value corresponding to first-component antibodies (FI_1_) over the FI_TOT_ of the sample, %FI_1_, was a measure of the proportion of high-avidity HBsAg-specific antibodies. W_1_ was proportional to the full width at half maximum (FWHM) of the capture profile of the first-component antibodies and represented their binding avidity. W_1_ values <5 (corresponding to K_D_ < 10^−10^ M in Biacore measurements), were arbitrarily defined as ‘high-avidity’^24,25^ (submitted manuscript). Thus, the FI-based (FI_1_, FI_TOT_, %FI_1_) and W_1_ parameters represented quantitative and qualitative approaches, respectively, to characterize the avidity of the main antibody populations in polyclonal serum.

### AS elicit higher proportions of high-avidity antibodies than Alum after antigen recall

We then investigated the adjuvants’ capacities to enhance the relative proportion of high-avidity antibodies produced after each antigen encounter, by comparing %FI_1_ kinetics between groups at D30, D60, D360, and D390. To optimally appreciate any differences in avidity, we performed these analyses on subjects of the cohort subset with CLIA antibody concentrations and FI_TOT_ values higher than or equal to the respective assay cut-offs, on at least one of these timepoints (*N* = 95).

**Fig. 4:**
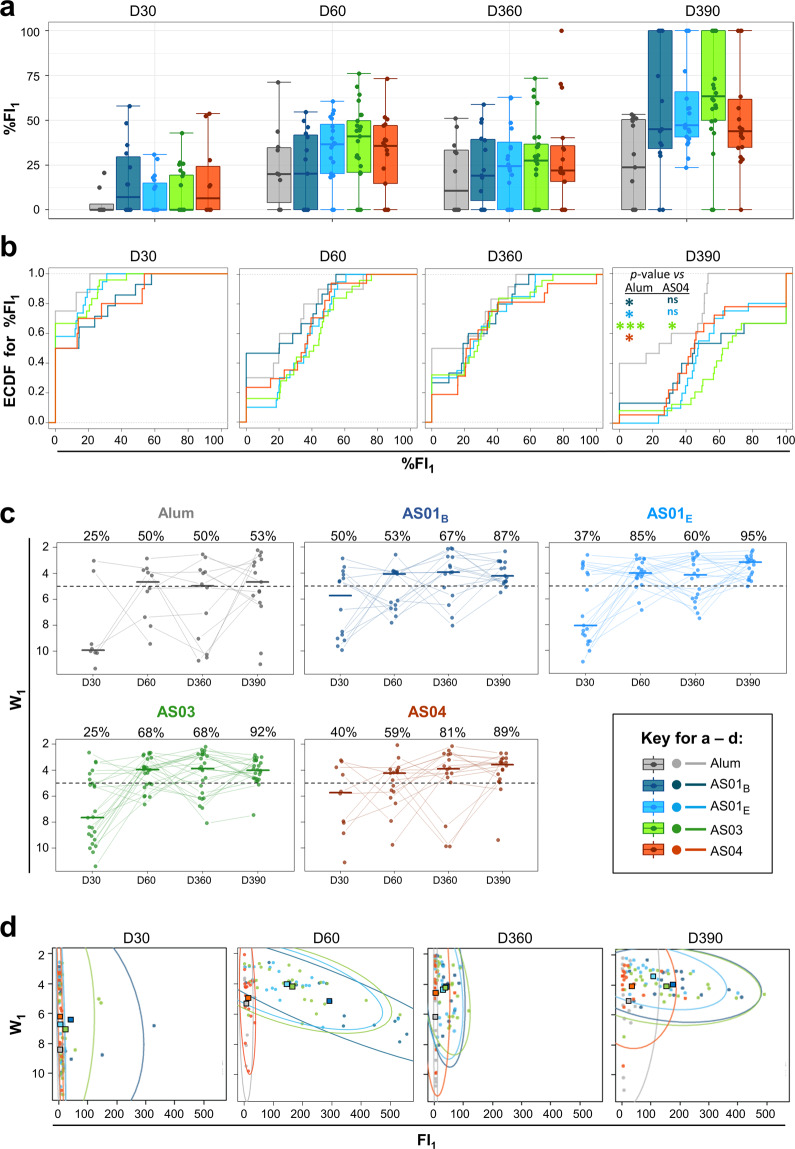
Adjuvant Systems stimulate avidity maturation before and after antigen recall. Data for the Avidity cohort (*N* = 95) are color-coded by adjuvant group according to the key in the figure’s center. FI_1_, fluorescence intensity attributable to first-component antibodies. **a** Frequencies of first-component antibodies in the total HBsAg-specific antibody population (%FI_1_) are shown. Boxplots represent medians, interquartile ranges (IQR), and ±1.5 IQR. **b** Empirical cumulative distribution function (ECDF) curves of the subjects are plotted against %FI_1_ data. For a given value of *t* %FI_1_, ECDF(*t*) represents the fraction of subjects in the respective group with %FI_1_ ≤ *t*. Groups for which the curve is shifted to the right are composed of subjects with increased %FI_1_ values. Curves were compared by a one-sided Kolmogorov-Smirnov test without correction for multiplicity. Asterisks indicate levels of significance by color-coded group (*p < 0.05, **p < 0.01, ***p < 0.001). **c**. Avidity of first-component antibodies (W_1_) is shown. Each symbol represents a subject. Gray lines connect results from the same subject. Horizontal solid lines represent group medians. Dotted lines represent the threshold dividing subjects with either high-avidity (W_1_ < 5) or medium-to low-avidity (W_1_ ≥ 5) antibody profiles. Numbers above the plots represent percentages of subjects in the group with high-avidity profiles, by timepoint. **d**. FI_1_, W_1_ plots show 95% confidence intervals **(**ellipses), group means (squares), and individual subjects (dots). Ellipse extensions reflect either higher numbers of high-avidity antibodies (shift on the *x*-axis to the right), or increased antibody avidity (upward shift on the *y*-axis). Upper-right corners represent the optimal situation: a high abundance of high-avidity antibodies.

Median proportions of high-avidity antibodies post-dose 1 (D30) were either slightly increased (~5% FI_1_; AS01_B_, AS04) or at baseline (other adjuvants; Fig. 4a). The proportions increased post-dose 2 (D60) to circa 20% (AS01_B_, Alum) or 35–45% (other AS) of the total amount of bound antibodies. One year post-vaccination (D360), median %FI_1_ levels were slightly reduced, and distributions were largely comparable between groups. However, after the revaccination (D390), a dichotomy was observed between Alum and the AS (medians: ~25% vs*.* 45–65%, respectively), with a tendency towards a higher median for AS03. In addition, higher-than-median %FI_1_ levels tended to be more frequent for AS01_B_ or AS03 as compared to the other adjuvants (Q3: 100 vs. 50–65%, respectively).

We then plotted the distributions of subjects (*y*-axis) with levels less than or equal to a given %FI_1_ value (*x*-axis), using Empirical Cumulative Distribution Function (ECDF) curves (Fig. 4b). Across groups, at least half of the subjects displayed baseline %FI_1_ at D30. These proportions were reduced at D60 (though only slightly for AS01_B_) with comparable distributions between groups. The latter was also the case at D360, though baseline values were detected for half of the Alum recipients, and in ~20–35% of AS recipients. Stronger contrasts between the distributions for Alum and the AS were seen at D390. While for Alum the D360 and D390 distributions were comparable, in the AS groups more recipients displayed increased %FI_1_ values at D390 relative to D360 (i.e., curves shifting to the right). This trend appeared most evident in the AS03 group. For example, proportions of subjects with FI_1_ ≤ 60% were circa 0.4, 0.5, and 0.7 for AS03, AS01_B_, and AS01_E_/AS04, respectively, but 1.0 for Alum. These trends were statistically confirmed (Kolmogorov-Smirnov test without correction for multiplicity) by significant intergroup differences, detected only at D390. These differences comprised higher values for AS01_B_, AS01_E_, AS03, and AS04 than for Alum (*p-*values: 0.04, 0.01, 9 × 10^−5^, and 0.02, respectively), and higher values for AS03 as compared to AS04 (*p*-value = 0.04).

Overall, the use of an AS instead of Alum for the first two vaccinations appears to have functionally altered memory B cells, such that higher proportions of high-avidity antibodies are produced upon antigen recall in vivo. This trend was particularly manifest for AS03.

### Stronger promotion of avidity maturation by AS than by Alum

We next compared the extents of avidity maturation between groups based on the kinetics of W_1_, used as a proxy for the quality of different responding memory B cells after the adjuvanted vaccinations or antigen recall (Fig. 4c). Compared parameters included the group medians of W_1_ and the frequencies of ‘high-avidity’ (W_1_ < 5) subjects. At D30, at least half of the subjects across groups displayed moderate-to-low avidity profiles, and the largest differences were detected between Alum and AS01_B_ (W_1_ medians [‘high-avidity’ subject frequencies]: 9.9 [25%] vs*.* 5.7 [50%], respectively). After the second vaccination (D60), avidity increased in all five groups (4.0–4.7 [50–85%]) but was lowest in the Alum group. At D360, this divergence was amplified, as avidity was high across the AS groups while remaining at the threshold in the Alum group (3.9 or 4.1 [60–81%] vs. 5.0 [50%], respectively). Post antigen recall (D390), the increase in the frequency of ‘high-avidity’ subjects was greater for AS01_B/E_ and AS03 than for AS04 (20–35 vs*.* 8%, respectively). However, the vast majority of AS recipients exhibited high-avidity profiles, while in the Alum group levels barely changed upon the antigen recall (3.1–4.2 [87–95%] vs*.* 4.7 [53%], respectively). This suggests that avidity maturation upon antigen recall is more strongly promoted by an AS than by Alum.

Finally, we integrated the qualitative and quantitative avidity characterizations. W_1_, FI_1_ distribution plots (Fig. 4d) revealed that post-dose 1 avidity levels were similar between the AS and slightly lower for Alum (means [squares in the plots] W_1_: 6–7 and ~8.5, respectively). Upper levels of the 95% confidence intervals of FI_1_ (UL; ellipses) were greater for AS01_B_ and AS03 than for the other adjuvants (~150 and ~300 vs. <50, respectively). However, only a few subjects (dots) receiving AS01_B_ and AS03 displayed high FI_1_ levels. At D60, avidity increased in all groups (means W_1_: 4–6), confirming that avidity maturation was enhanced by the second dose. Of note, distributions still overlapped for AS04 and Alum, at low (<50) FI_1_ levels. By contrast, distributions for AS01_B/E_ and AS03 were expanded (UL > 450) and encompassed substantially more ‘high-avidity’ subjects as compared to Alum and AS04. At D360, avidity levels remained higher in the four AS groups than in the Alum group, though numbers of ‘high-avidity’ subjects markedly contracted in the AS01_B/E_ and AS03 groups. Post-revaccination (D390), avidity levels increased across groups, and the difference in avidities levels for Alum vs. the AS was maintained (means W_1_: ~5 vs*.* 3.5–4, respectively). As at D360, more AS01_B/E_ or AS03 recipients than AS04 recipients displayed high-avidity antibody profiles (UL: ~500 vs. ~200_,_ respectively). This suggests that while all AS induce similar extents of avidity maturation, the secreted quantity of high-avidity antibodies is lower for AS04 than for AS01 and AS03. This was consistent with our conceptual model of different levels of T cell help provided by these adjuvants.

## Discussion

The effects of adjuvant composition on durability and functionality of immune memory that can be boosted by antigen recall, remain underinvestigated. We compared long-term immunogenicity, recall responses, and antibody avidity maturation between HBsAg vaccines formulated with adjuvants used in licensed vaccines. Our analyses revealed the following. First, despite inducing different peak antibody, memory B cell, and CD4^+^ T cell responses, the adjuvants’ compositions impacted neither the durability of these responses one-year post-vaccination, nor the response magnitudes upon antigen recall (though inter-subject variability in anamnestic antibody responses seemed greater for AS04). Second, inter-group differences in the extent of avidity maturation suggested a benefit of using an AS over Alum. Third, differences *between* the four AS were subtler: we observed trends for higher relative proportions of high-avidity antibodies upon antigen recall for AS03, and for lower absolute amounts, but similar relative proportions of high-avidity antibodies for AS04.

**Fig. 5:**
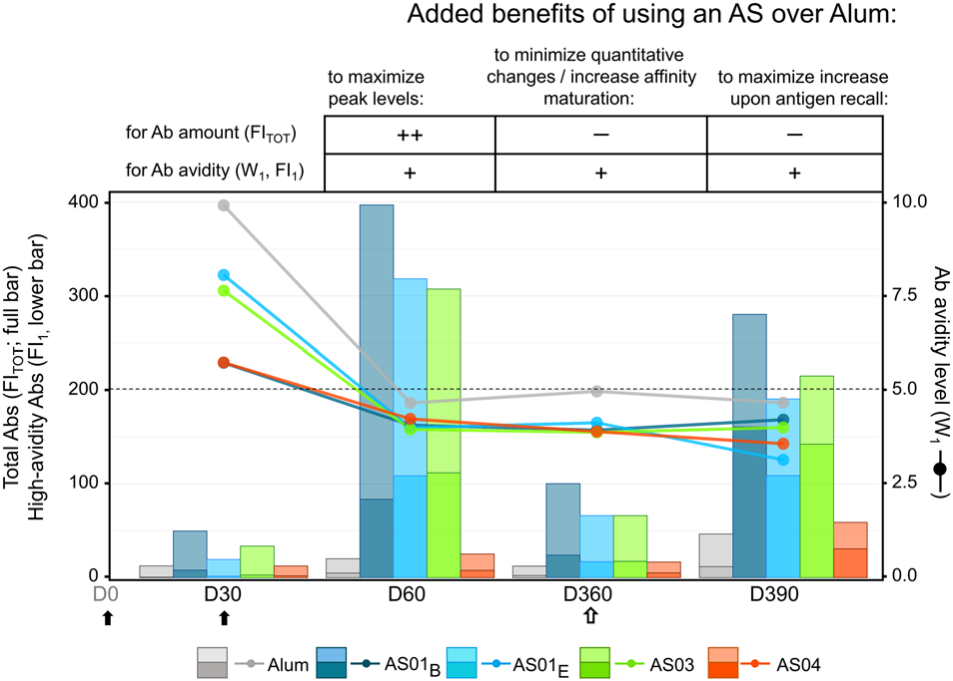
Benefits of Adjuvant Systems over Alum for vaccine antibody responses. Schematic presentation of the benefits of using an Adjuvant System (AS) over Alum to formulate HBsAg vaccines is presented for the different aspects of the antibody (Ab) response. The immunization schedule comprised two primary vaccinations administered at day (D)0 and D30 (solid arrows) and a non-adjuvanted antigen recall (open arrow) administered at D360. The table summarizes the expected added benefits of the adjuvant choice (an AS or Alum) for enhancement of quantitative and qualitative aspects of the response at different timepoints post-vaccination or after antigen recall; ‘++’ and ‘+’ denote significant and moderate-to-slight enhancement, respectively, and ‘–’ denotes no clear benefit, i.e., similar proportional decline/enhancement for AS and Alum. The graph shows the abundance of HBsAg-specific Abs (total fluorescence intensity [FI_TOT_]; full bars), the relative abundance of high-avidity specific Abs (fluorescence intensity of the first component Abs [FI_1_]; the bottom part of bars), and Ab avidity levels (W_1_; lines/symbols). Bars, lines, and symbols are color-coded according to the adjuvant groups presented below the graph. The horizontal dotted line represents the threshold for high avidity, with high-avidity profiles defined as W_1_ < 5.

The persistence of humoral and cellular responses seen with all five adjuvants indicates that cell-mediated immune memory was induced and maintained, and resulted in ongoing antibody production. This aligns with data for other candidate or licensed vaccines against herpes zoster (AS01), pandemic influenza (AS03), HPV (AS04), or HBV (Alum)^27,28,35–42^. Remarkably, only minor differences in antibody longevity and recall responses were seen between the adjuvants, which may align with the similar titer half-lives for MPL/Alum or Alum alone in macaques^43^. The similarity observed here contrasts with the considerable differences in innate response magnitudes between these adjuvants^12,20,44^. These responses translated into peak adaptive responses that differed vastly between AS01_B/E_ or AS03 vs*.* AS04, and between AS04 and Alum^12,21^. Long-term maintenance of serum antibody levels is mediated by continuous antibody secretion by terminally differentiated, long-lived PCs, a proportion of which will reside in the BM^45^. We hypothesize that, due to the AS01/AS03–AS04/Alum divergence in innate immune induction, both the circulating B cells and the fractions of these cells migrating to the BM, will be more numerous for AS01/AS03 than for AS04/Alum. Once PCs reach the BM, various immune stimuli are needed for the transcriptomic and metabolic changes determining their differentiation into long-lived PCs^46^. However, the extent to which these processes can be improved by adjuvants remains to be determined, and our D360 data suggest that the behavior and longevity of the BM PCs are also independent of the original innate immune activation. Furthermore, the similar antibody recall responses for the five groups suggest that also boost ability of memory B cells was independent of the original innate signal mediated by these adjuvants. This similarity could be caused by comparable levels of antibody production for the adjuvants, due to a hypothetical maximum production threshold per B cell. Further research to test these hypotheses could focus on T_FH_ cells in the draining lymph node, B cell receptor (BCR) repertoires, and specific antibody subtypes, all of which were identified as correlates of long-term immunity in mice^47^ (though this may not be fully predictive for primates^43^). Our findings may also be supported by assessing BM B cells in animal models. Finally, these effects can be antigen-dependent and at least partially specific to HBsAg. Indeed, specific antibody responses to RTS,S/AS01_E_ vaccine (circumsporozoite [CS] malaria antigen on an HBsAg carrier) were significantly less durable for CS than for HBsAg^48^, despite the induction of CS-specific memory B cells and T-helper cells^49,50^. We conclude that from a quantitative perspective, the benefit of using a specific adjuvant or AS lies in maximizing the peak response, to prolong the duration of protection when immunity wanes over time (Fig. 5).

Primary vaccination with TLR or oil-in-water-adjuvants elicits high-avidity antibodies –as demonstrated for these AS combined with other antigens^10,18,41,51,52^—and the induced memory B cells undergo further maturation and differentiation upon antigen recall. Minor differences in quantitative antibody persistence were detected between the adjuvants. Furthermore, affinity maturation parameters (i.e., the W_1_-based avidity levels, numbers of subjects with a high-avidity response, and %FI_1_-based proportions of high-avidity antibodies) appeared to differ when comparing the AS with Alum. Indeed, in the AS groups, levels of at least one of these parameters increased after each antigen exposure, consistent with murine data showing that repeated immunization is required for somatic mutation and avidity maturation^53^. The increase in avidity over time suggests that the robust innate immune stimulation provided by AS01_B/E_ and AS03 had a long-term effect on the quality of memory B cells and CD4^+^ T cells. We hypothesize that this caused these cells to evolve after each exposure, magnifying the contrasts between vaccine arms over time. The difference after revaccination between the AS vs. Alum may be due to imprinted differences in memory B cells or BCR repertoires, as a result of different levels of T cell help received by antigen-specific B cells and plasmablasts. This bias towards higher-avidity antibodies for AS recipients suggests that the antigen recall preferentially stimulated memory B cells that had received higher-quality T cell ‘help’ mediated through innate stimuli from the AS-containing vaccines. A non-exclusive alternative mechanism is that B cells with the highest avidity are most successful in capturing and subsequently presenting the antigen (MHC-peptide complex) to T_FH_ cells and that such cells thus receive the strongest expansion and maturation signals.

Interestingly, AS04 induced an avidity maturation pattern similar to that seen for AS01 and AS03, despite being less potent in terms of absolute quantities of high-avidity antibodies and adaptive cells. Similarly, avidity for the vaccine antigens increased over time and/or with the number of vaccinations following AS04-adjuvanted HPV vaccination^17,41,54^. The trend for higher proportions of high-avidity antibodies after the antigen recall for AS03 relative to the other adjuvants was unexpected, given the similar innate and transcriptomic responses post-dose 2 previously detected for AS01 and AS03^12,20^. This is of interest when compared with observations in infant macaques, where antibody avidity to the (HIV) vaccine antigen tended to be *lower* with the oil-in-water adjuvant MF59 as compared with AS01^55^. Possibly AS03 delivers a unique signal to the responsible APCs, B cells, and CD4^+^ T cells that went undetected in the previous analyses, and in which α-tocopherol present in AS03 plays a role^15^. The collective data highlight the need to further our understanding of the correlation between adjuvant-induced innate responses and antibody quality. Of note, AS03 is currently being evaluated for the benefit of different COVID-19 vaccine candidates in development^56–59^. The importance of antibody avidity is further illustrated by the observation that increased mAb affinity mediated broader protection against SARS-coronavirus strains^60^.

The conclusion that low-dose antigen recall in the absence of adjuvant mediated both qualitative and quantitative immune enhancements is of particular interest considering previous evaluations of fractional-dose RTS,S/AS01 vaccine, or AS03-adjuvanted pandemic influenza vaccines. These studies demonstrated that, relative to the full-dose vaccines, fractional-dose *priming* vaccinations induced comparable neutralizing titers^61^, and fractional-dose *booster* vaccinations induced increased levels of antibody avidity and somatic hypermutation frequency in B cells^18,19^. Possibly this is explained by murine data obtained after immunizations with limiting concentrations of protein antigen^62–64^. Under these circumstances, antigen-specific B cells, acting as APCs, mediated priming of circulating T cells to deliver T cell help, leading to competitive exclusion of B cells with lower specificities, and thus to higher proportions or higher-avidity antibodies. By contrast, B cells with APC abilities were not required using high antigen doses. Overall, the responses to non-adjuvanted antigen recall indicate that an adjuvant may not be required to obtain a robust response to a booster dose following primary vaccinations. Besides the obvious advantages with respect to antigen sparing and adjuvant sparing, this concept may also be of interest in specific populations or settings in which administration of adjuvants may be a theoretical concern. Particularly in these cases, decisions on booster composition should be grounded in careful benefit/risk analyses. This is highlighted by the link between innate immune responses or prevaccination B cell phenotypes, and reactogenicity, as detected for some AS-adjuvanted vaccines^12,44,65^. Currently, informed decision-making on this issue is hampered by the lack of control groups receiving adjuvanted reduced-antigen boosters.

It is generally accepted that CD4 helps stimulates affinity maturation^23^, with T_FH_ cells as the implicated T cell subset. The observation that the addition of MF59 led to increased T_FH_ responses in human vaccinees^66^ suggests that a similar mechanism operates here, and could explain how AS drive increased avidity. We acknowledge that our study was limited by a lack of specific markers (i.e., CXCR5, PD1, and BCL6) and timepoints (e.g., D7 post-vaccination^67^) to detect circulating T_FH_ cells. This prevents determining whether the qualitative enhancement by AS was due to increased T_FH_ cell activation and promotion of germinal center reactions. Nevertheless, this notion seems to be supported by the T_FH_-cell responses detected in lymph nodes of AS03-treated mice, and in the blood of human recipients of AS01-adjuvanted malaria vaccines^67,68^. Further analysis of our dataset demonstrated that circulating CD40L^+^ CD4^+^ T cell levels at D360 neither correlated with the increase in antibody titers post-revaccination (data not shown) nor with peak titers^21^. Moreover, all five vaccine arms displayed similar functional cytokine profiles of responding CD4^+^ T cells^21^. Therefore, our results merit deeper T cell phenotyping and TCR repertoire analyses. In addition, BCR repertoire sequencing (as performed for HBsAg/Alum and AS03-adjuvanted influenza vaccines^11,69^), plasmablast identification, and deep profiling of the functionalities of the humoral response such as antibody isotype distribution and fine-specificity (manuscript in preparation) may further elucidate underlying immune mechanisms.

Finally, our innovative approach to measuring the avidity of polyclonal serum antibodies is promising in the context of vaccine clinical trial settings, due to the high-throughput set-up^24,25^ (submitted manuscript). It offers considerable advantages over existing avidity assays, which either provide avidity measures averaged over all mAb populations (chaotropic ELISA, biolayer interferometry), are time-consuming (surface plasmon resonance), or can be biased by antibody specificity (chaotropic ELISA^70^).

Altogether, despite inducing quantitatively dissimilar levels of immune responses, differently composed Adjuvant Systems appear to have similar abilities to induce persistent immunity and robust responses after antigen recall. Relative to AS01_B/E_ and AS03, AS04 elicited fewer high-avidity antibodies but comparable avidity maturation patterns. These compelling data can be exploited in vaccine design, formulation, and dosing.

## Methods

### Ethical statement

The observer-blind, randomized, controlled trial (ClinicalTrials.gov identifier: NCT00805389) was registered on December 9, 2008, and conducted from December 2008 to July 2011. The protocol was approved by all institutional Ethics Committees and was conducted in accordance with the Declaration of Helsinki and Good Clinical Practice guidelines. Written informed consent was obtained from each participant before trial participation.

### Study design

Participants were healthy HBV-naïve men and women aged 18–45 years, who were randomized (1:1:1:1:1) to receive two doses of 20 μg HBsAg vaccine adjuvanted with AS01_B_, AS01_E_, AS03, AS04, or Alum^21^. AS01_E_ contained half the MPL and QS-21 quantities present in AS01_B_. QS-21 refers to *Quillaja saponaria* Molina, fraction 21 (Licensed by GSK from Antigenics LLC, a wholly-owned subsidiary of Agenus Inc., a Delaware, USA corporation). Participants were vaccinated at D0 and 30 and received a challenge dose with 5 μg non-adjuvanted HBsAg antigen at 1-year post-dose 1 (D360). They were followed until one-month post-challenge (D390).

As pre-specified analyses, persistence and boost ability of the adaptive immune response were evaluated in the Booster ATP immunogenicity cohort (‘Booster cohort’; *N* = 265), and in a cohort subset (*N* = 99). This subset comprised only the subjects selected for microarray analyses, as reported elsewhere^20^. The AS01_B_, AS01_E_, AS03, AS04, and Alum groups contained 55, 52, 54, 54, and 50 participants in the Booster cohort, and 15, 20, 25, 18, and 21 participants in the cohort subset, respectively. Blood samples were collected at prevaccination (D0), 1 month after the first dose (D30), and 2 weeks, 1 month, and 5 months after the second dose (D44, D60, and D180, respectively). Blood samples were also collected before and two weeks after the antigen recall (D360 and D374), and, for analyses of anti-HBsAg antibodies only, one month after the antigen recall (D390). Adaptive immune responses up to D60 have previously been described for a larger cohort (*N* = 293 for antibodies and memory B cells; *N* = 599 for CD4^+^ T cells)^21^.

Antibody avidity (a post-hoc analysis) was evaluated at D30, D60, D360, and D390. Analyses were performed for the Booster cohort subset (*N* = 99; described above), excluding three subjects who were (CLIA) seronegative on all four timepoints (see Supplementary Fig. 4c). This left 96 subjects in the initial analysis. One subject was subsequentially excluded due to exhibiting FI_TOT_ values below the Gyrolab assay cutoff (specified below) on all four timepoints. Therefore, all other avidity analyses (see Figs. 4 and 5, and Supplementary Fig. 4 a/b) were performed on the remaining 95 subjects with both the CLIA antibody concentration and FI_TOT_ value ≥ respective assay cut-offs, on ≥1 of the four timepoints. These 95 subjects are elsewhere in this article referred to as the ‘Avidity cohort’ (*N* = 15, 20, 25, 18, and 17, in the AS01_B_, AS01_E_, AS03, AS04, and Alum groups respectively).

### Quantitative immunogenicity assays

As described previously^21^, total anti-HBsAg immunoglobulin (Ig) concentrations were measured using a commercial CLIA with a cutoff of ≥6.2 mIU/mL. HBsAg-specific memory B cell frequencies, and frequencies of HBsAg-specific CD4^+^ T cells expressing at least CD40L, were quantified by ELISPOT assay and intracellular cytokine staining (ICS) assay, respectively^21^. Frozen peripheral blood mononuclear cells were used in both assays. ELISPOT data are expressed as numbers of HBsAg-specific IgG-producing memory B cells per million IgG-producing memory B cells. ICS data are expressed as numbers of HBsAg-specific CD4^+^ T cells expressing at least CD40L, per million CD4^+^ T cells, after subtracting the background values. The activation marker CD40L was selected because CD40L^+^ CD4 T cells likely contain T_FH_ subsets, as CD40L signaling leads to leads to B cell activation^8^. The use of this marker also allowed detection of HBsAg-specific CD4^+^ T cells not producing the measured cytokines (IL-2/IFN-γ/TNF-α), which represented a substantial proportion (around one-third) of the CD4^+^ T cell response^21^. Descriptive statistical analyses were performed using SAS software (SAS Institute, Cary, NC).

### Avidity characterization

Antibody avidity for HBsAg was quantified using an adaptation of a high-throughput methodology initially developed to quantify mAb or Fab affinity^24,25^ (submitted manuscript).

Briefly, FI distributions (averaged capture profiles) obtained by Gyrolab-miniaturized immunoassay^34^ were subjected to mathematical modeling using a deconvolution algorithm. The methodology allowed separating the antibody populations in a serum sample into a first and second component containing the higher-avidity and medium-to-low-avidity antibodies, respectively. The separate steps are outlined below (“Generation of input data by Gyrolab immunoassay”, “Data analysis pipeline to estimate the avidity of pAb solutions” and “Application to the adjuvant group comparison”).

### Generation of input data by Gyrolab immunoassay

Antibody avidity for HBsAg in post-vaccination sera was quantified using an adaptation of a high-throughput methodology initially developed to quantify mAb or Fab affinity in out-of-equilibrium solutions (refs. ^24,25^; submitted manuscript). The methodology entails the mathematical modeling of fluorescence intensity (FI) distributions obtained by Gyrolab microfluidic ligand-binding immunoassay^34^. In the current Gyrolab analysis, the capture reagent was biotinylated HBsAg diluted in PBS/Tween20 (0.01%), used at 100 µg/mL. Goat anti-human Fcγ-fragment specific IgG (Jackson ImmunoResearch; #109-606-170) at 25 nM was used as detection reagent and Rexipp F buffer (Gyros Protein technologies) as diluent. Control (buffer only) samples were run in parallel to determine experimental background signals. Data were analyzed according to the Gyrolab three-step method (capture, analyte, detection) using Gyrolab Bioaffy 200 CDs and Gyrolab Viewer software. Each serum sample in the analysis (*N* = 342) was run at least 5 times at a 1:40 dilution. This dilution was selected based on dose-response curves generated for 43 randomly chosen serum samples to select the range of linearity (i.e., the range where concentration is linearly proportional to the FI signal). Two-dimensional capture profiles were obtained by integrating the FI signals along the longitudinal coordinate, such that the FI value (*y*-axis) as a function of the radial coordinate of the antigen-saturated capture column (*x*-axis). From the individual capture profiles per run, one profile per sample was obtained by computing the mean and 95% confidence intervals (CI) from the distribution of FI values obtained by the repeated runs for each radial position. The resulting averaged capture profile with 95% CI (‘pAb profile’) was the input of the algorithm to define avidity (see Section “Data analysis pipeline to estimate the avidity of pAb solutions”).

As reported elsewhere (refs. ^24,25^; submitted manuscript), capture profiles for mAb solutions can be described by fitting an approximated Landau probability distribution^71,72^ *L(x)* to the data, with a correction for background signals:

1 $$L\left( x \right) = y_0 + A \cdot \exp \left[ { - \frac{1}{2}\left( {\frac{{x - m}}{W} + \exp \left( { - \frac{{x - m}}{W}} \right)} \right)} \right]$$

where *m* is the radial coordinate of the FI peak, *W* is the affinity score, *A* is a normalization parameter, and *y*_*0*_ is the background signal. *W* is proportional to the curve’s full-width-at-half-maximum, and *A* is proportional to the FI peak value. In addition, the starting point of a profile was defined as the radial coordinate where FI ≤ 1% of FI peak and represented the lowest coordinate corresponding to a binding signal rather than to a background signal.

### Data analysis pipeline to estimate the avidity of pAb solutions

Because a pAb profile constitutes a mix of the profiles of populations of mAb-like clones of similar avidity (‘components’), the avidity of a sample could be estimated by applying a deconvolution algorithm to the pAb profile. The aim was to identify the number of components (1, 2, or 3), quantify the avidity parameters for each component, and identify the highest-avidity component. The analysis pipeline is summarized in Supplementary Fig. 5. To enhance the variability of the repeated profiles, 100 bootstrap profiles were created from each pAb profile. This was done by drawing for each radial position, an FI value from a uniform distribution, with the extreme FI values corresponding to the 95% CI of the pAb profile.

Estimation of the *m* value of the first component (*m*_1_) was required for a correct regression. This was performed by measuring the pAb profile’s starting point, *SP*_*MIX*_, directly from the profile, and by estimating a variable *q* from the bootstrap profiles. Because the locations within the lattice locations differ between different CDs due to set-up variability, *q* is a CD-specific parameter. The following assumptions were made:

- The area-under-the-curve (*area*) for mAbs relates to *A* and *W*, as: *area* = *A* × *W* × *√ (2π)*;
- There is a linear relationship between *m* and *W*, namely: *m* = *s* × *W* + *q*, where *s* = 1.47. Using this equation with *W* and *q* as free parameters, a two-component nonlinear least square (NLS) regression was performed of all bootstrap profiles generated for the same CD. A mean *q* was then computed from the obtained distribution of *q* values;
- For mAb profile, the starting point (SP_MONO_) relates to *W* as: SP_MONO_ = 2.634 × *W*, and the starting point for its first component, SP_MONO1_ relates to the SP_MIX_ as:

SP_MONO1_ = *1.038* × SP_MIX_.

Using the mean *q* value (see b), *m*_*1*_ is then described as:

2 $$m_1 = \frac{{\frac{{2.634}}{s}.q - 1.038.{\mathrm{SP}}_{{\mathrm{MIX}}}}}{{\frac{{2.634}}{s} - 1}} - {\mathrm{bias}}$$

where ‘bias’ represents a constant correction term to minimize the error between expected and real values, equal to −3.

In the “model selection” step the number of components of the pAb profile is selected. Based on the *L(x)* distribution (see Section “Generation of input data by Gyrolab immunoassay”), a 1-component, a 2-component, and a 3-component NLS regression were performed for each pAb profile, using for the multi-component regressions the *m*_*1*_ value determined in Step 1. The three regressions were compared based on their Bayesian Information Criterion (BIC) score. The model with the lowest score was selected, provided the following two threshold criteria set for *A* and FI were met: if for one component of a multi-component description, the FI value accounted for <10% of the total FI, and the *A* value was <10% of the *A* values of the other components, the description was discarded. This was done even if the associated BIC score was lower than that for a 1-component description, in order to prevent the algorithm from introducing small, non-biological Ab populations which would artificially improve the goodness-of-fit.

The selected model was applied to the 100 bootstrap profiles (using m_1_ in either case), yielding a set of *A*, *m*, and *W* values. From a mean *L(x)* regression for the selected component, the mean *A*, *m*, and *W* values were estimated for the pAb profile. Based on the obtained W values, the first or only component, and the second and third components (if detected), were defined as the high-avidity, medium-to-low avidity, and low-avidity components, respectively. Of note, across the current samples, the goodness-of-fit was consistently higher for the 1-component or 2-component descriptions than for the 3-component description, and the latter were therefore not included in the analyses. This can be explained by the fact that the samples were collected after vaccination, and contained few low-avidity antibodies.

### Application to the adjuvant group comparison

Comparisons between the adjuvant groups were based on the total FI value (area under the pAb profile; FI_TOT_) of the sample, representing its total amount of HBsAg-specific antibodies, and on the parameters of the first (or only) component. The latter parameters included the *W*_*1*_ affinity score as a qualitative criterion, and the percentage of the FI_TOT_ belonging to the first component (%FI_1_), i.e., the proportion of high-avidity antibodies of the total amount of specific antibodies, as a quantitative criterion. Due to the inability of the algorithm to analyze profiles close to the background noise, subjects with antibody capture profiles with an FI peak < technical cut-off of 0.15 (approximately 3 times the typical noise value) at all timepoints were excluded from the analyses. For each group, descriptive statistical analyses were performed on the W, FI, and %FI_1_ data, and ECDF curves (Fig. 4b) were generated. Proportions of subjects with a high-avidity (W_1_ < 5) antibody population with associated two-sided 95% Clopper-Pearson CI were calculated for each group and timepoint. ECDF values were compared using one-sided Kolmogorov-Smirnov tests without correction for multiplicity, as implemented in the ks.test() function in R software, v3.6.2 (R Foundation for Statistical Computing). Analyses and graphs were developed using RStudio version 1.1.463 (RStudio, Inc., Boston, MA).

### Reporting summary

Further information on research design is available in the Nature Research Reporting Summary linked to this article.

## Supplementary information

Supplementary Information

Reporting Summary

## Data Availability

GSK makes available anonymized individual participant data and associated documents from interventional clinical studies which evaluate medicines, upon approval of proposals submitted to www.clinicalstudydatarequest.com. To access data for other types of GSK sponsored research, for study documents without patient-level data, and for clinical studies not listed, please submit an inquiry via the website (ClinicalTrials.gov identifier: NCT00805389).

## Code availability

Full code used for the analyses presented in this manuscript is available upon request to the corresponding author.
