# Supplementary material for: Antibody avidity, persistence, and response to antigen recall: comparison of vaccine adjuvants
Source: NPJ Vaccines. 2021 May 21;6:78. doi: 10.1038/s41541-021-00337-0 (PMC8140094; doi:10.1038/s41541-021-00337-0)
Supplement: Supplementary file 1 — Supplementary Information [file 41541_2021_337_MOESM1_ESM.pdf]

Supplementary Figure 1. Association between the increase in antibody concentrations post-antigen recall, and memory B-cell frequencies pre-antigen recall. The plot presents the memory B-cell frequencies measured before the administration of fractional-dose HBsAg alone at Day (D)360, versus the increase in antibody levels upon the antigen recall (△ D390 – D360), as determined for the Booster cohort (N = 265). Negative values for △ D390 – D360 antibody concentrations were assigned a value of zero. Memory B-cell frequencies are expressed as numbers of HBsAg-specific immunoglobulin (Ig)G-producing memory B cells per million of IgG-producing memory B cells. Symbols are color-coded according to the adjuvant groups presented in the key. Each symbol represents an individual subject.

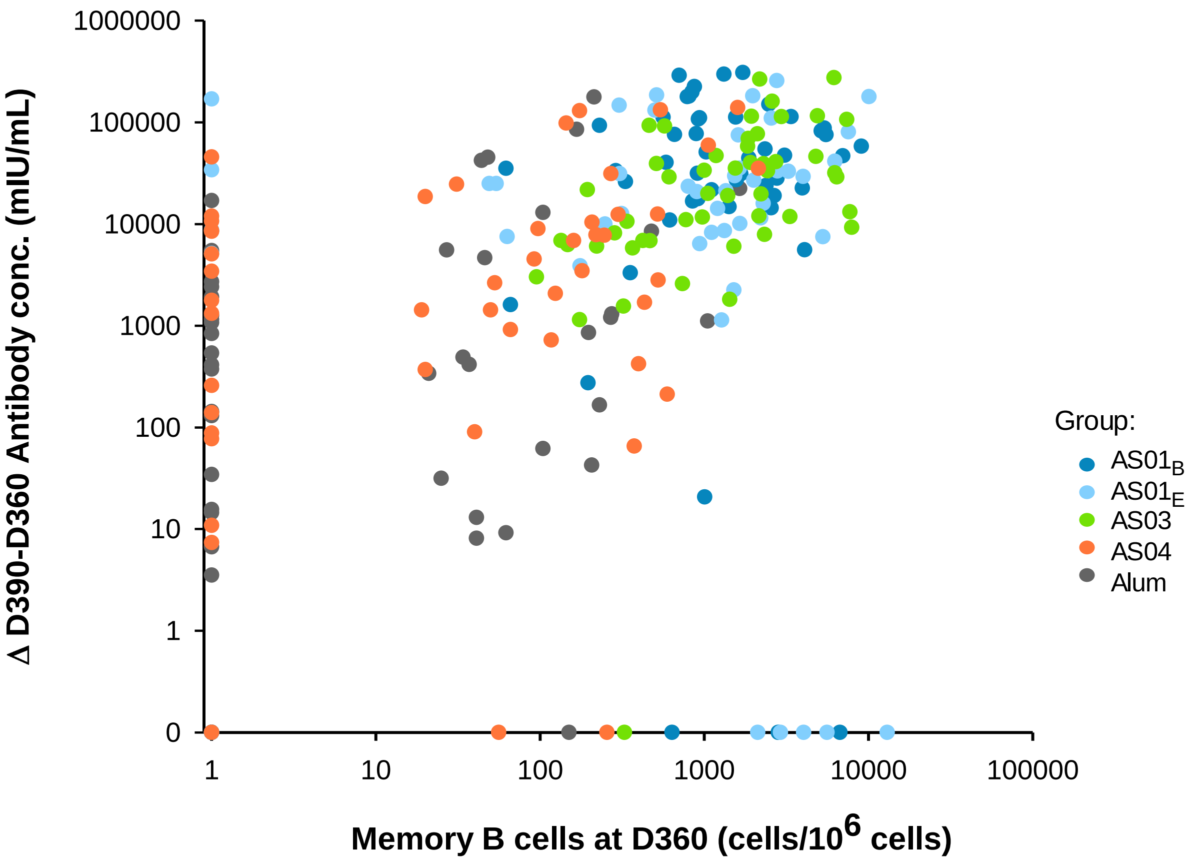

Supplementary Figure 2. Persistence and post-antigen recall responses of HBsAg-specific antibodies, memory B cells and CD4$^+$ T cells in HBsAg-seropositive subjects. Subjects received two doses of HBsAg vaccines adjuvanted with AS01$_B$, AS01$_E$, AS03, AS04 or Alum at Day (D)0 and D30, and a revaccination with non-adjuvanted HBsAg, using 1/4th of the adjuvanted antigen dose, at D360. Results are presented for a subset (N = 99) of the per-protocol Booster Cohort. Lines and symbols are color-coded according to the adjuvant groups presented in the key. Presented are the kinetics of geometric mean concentrations (GMC) of anti-HBsAg antibodies with 95% confidence intervals (CI; **a**), and the kinetics of geometric mean frequencies (GMF) of HBsAg-specific IgG-secreting memory B cells (**b**), or HBsAg-specific CD40L$^+$ CD4$^+$ T cells (**c**).

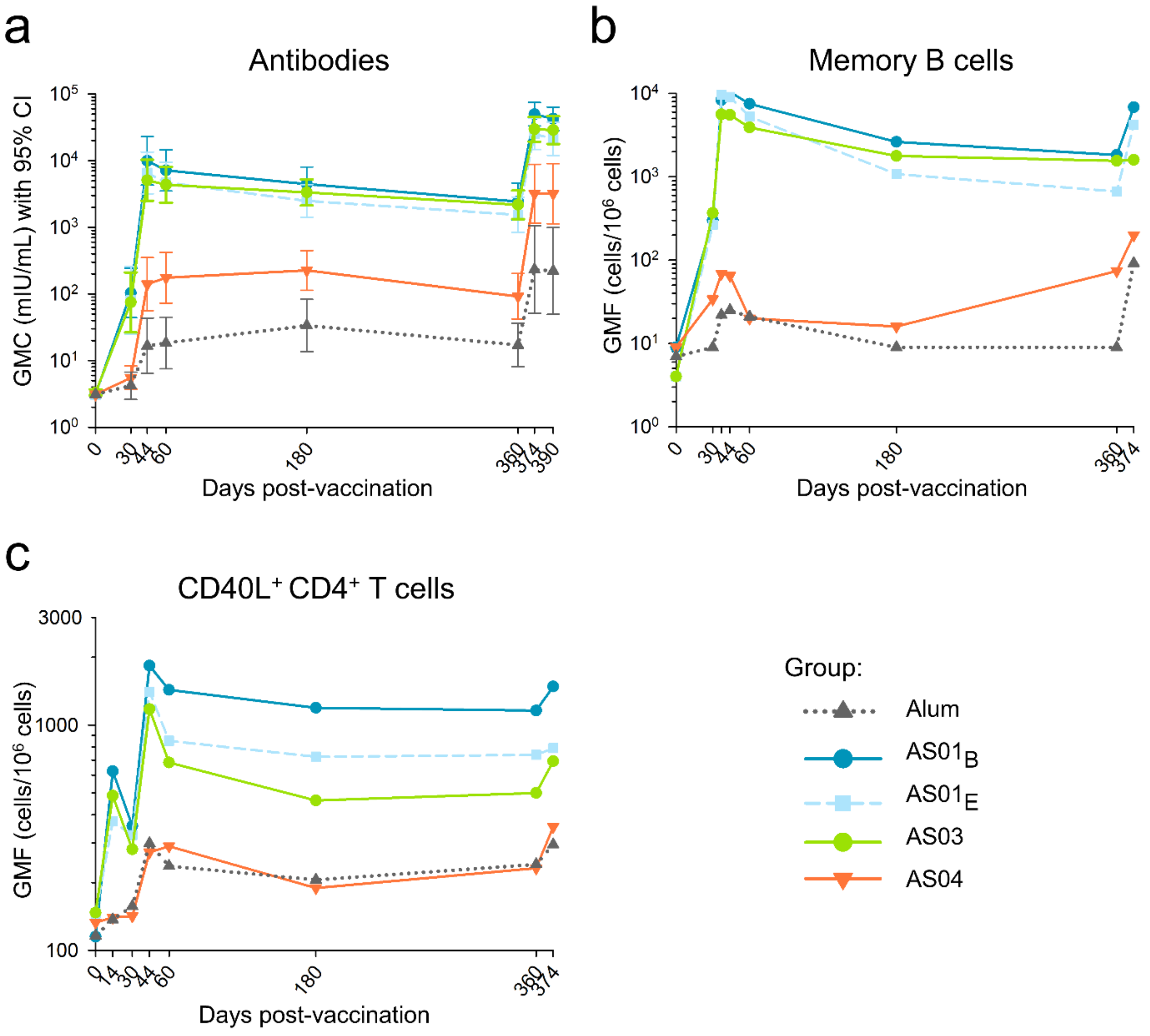

Supplementary Figure 3. Ratios of changes at the persistence time-point and post-antigen recall in HBsAg-specific antibody, memory B-cell and $CD4^+$ T-cell responses in HBsAg-seropositive subjects. Subjects received two doses of hepatitis B surface antigen (HBsAg) vaccine adjuvanted with $AS01_B$, $AS01_E$, AS03, AS04 or Alum at Day (D)0 and D30, and a revaccination with non-adjuvanted HBsAg at D360. Results are presented for a subset (N = 99) of the per-protocol Booster Cohort. Ratios of HBsAg-specific antibody concentrations, and of HBsAg-specific B-cell or $CD40L^+$ $CD4^+$ T-cell frequencies are presented for D360 over D60 (**a–c**), and for D390 or D374 over D360, i.e. post/pre-antigen recall (**d–f**). Boxplots represent medians, first and third quartiles, minima and maxima. Each symbol represents an individual subject.

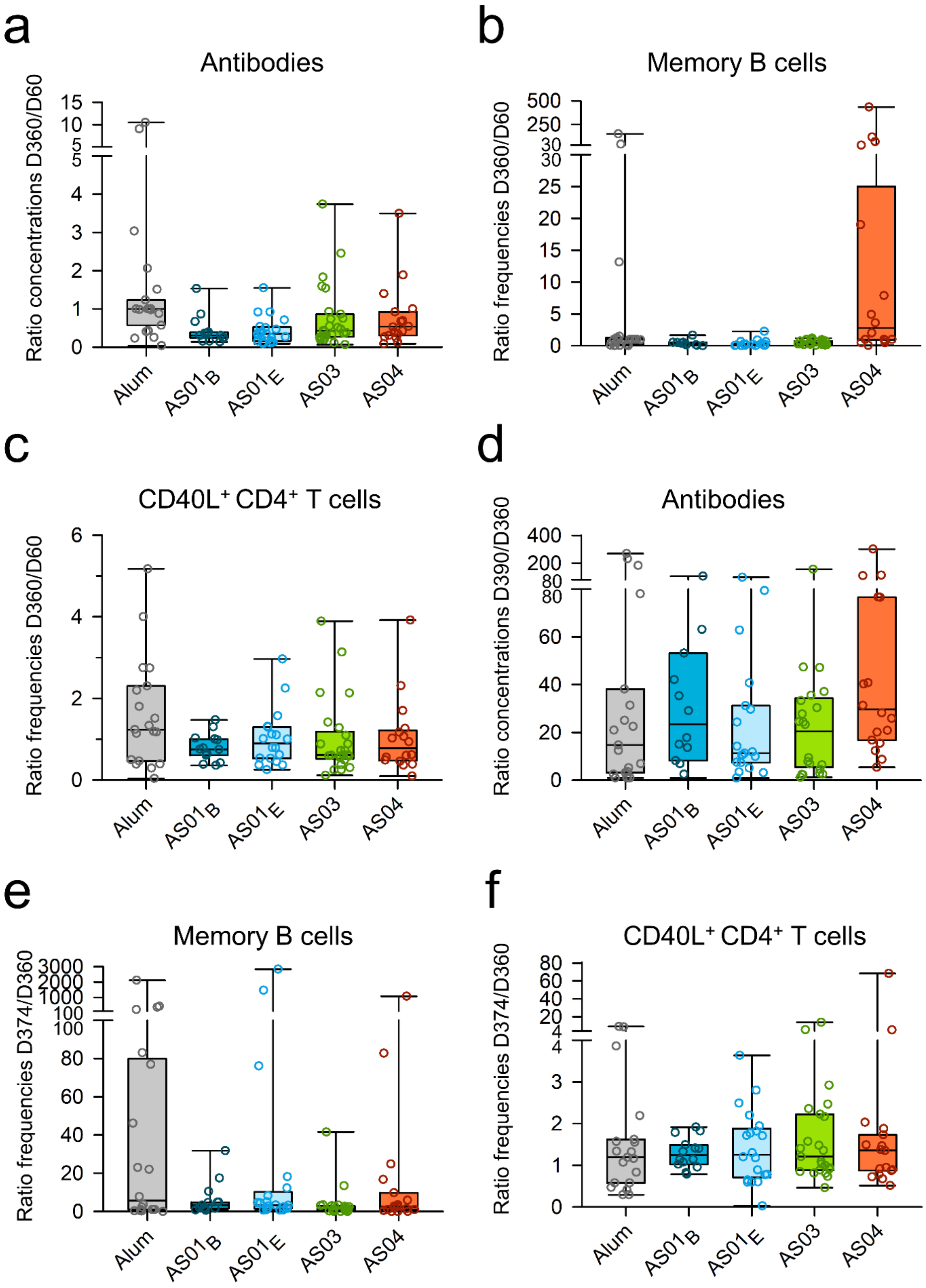
a
Antibodies
Ratio concentrations D360/D60
b
Memory B cells
Ratio frequencies D360/D60
c
CD40L⁺ CD4⁺ T cells
Ratio frequencies D360/D60
d
Antibodies
Ratio concentrations D390/D360
e
Memory B cells
Ratio frequencies D374/D360
f
CD40L⁺ CD4⁺ T cells
Ratio frequencies D374/D360
Alum
AS01B
AS01E
AS03
AS04

Supplementary Figure 4. Antibody concentrations correlate with total fluorescence intensities. Figure shows antibody concentrations obtained by chemiluminometric immuno-assay (CLIA) (**a**), total fluorescence intensities (FI_{TOT}) determined by Gyrolab microfluidic ligand-binding immunoassay (**b**) and the correlation between the two parameters at Day (D)60 (**c**). Analyses in panels **a** and **b** were performed for the Avidity cohort (N = 95) comprising subjects of the Booster cohort with values of at least the cut-offs for both assays on at least one time-point. The analysis shown in (**c**) was performed on a subset of the Booster cohort (N = 99). Boxplots represent medians and interquartile ranges (IQR), either +/− minima and maxima (**a**) or +/− 1.5 × IQR (**b**). Boxplots and symbols are color-coded according to the adjuvant group in the key. Each symbol represents an individual subject. Dotted lines represent values of half the CLIA cutoff (**a**) or the CLIA cutoff (**c**), i.e., 3.1 or 6.2 mIU/mL, respectively.

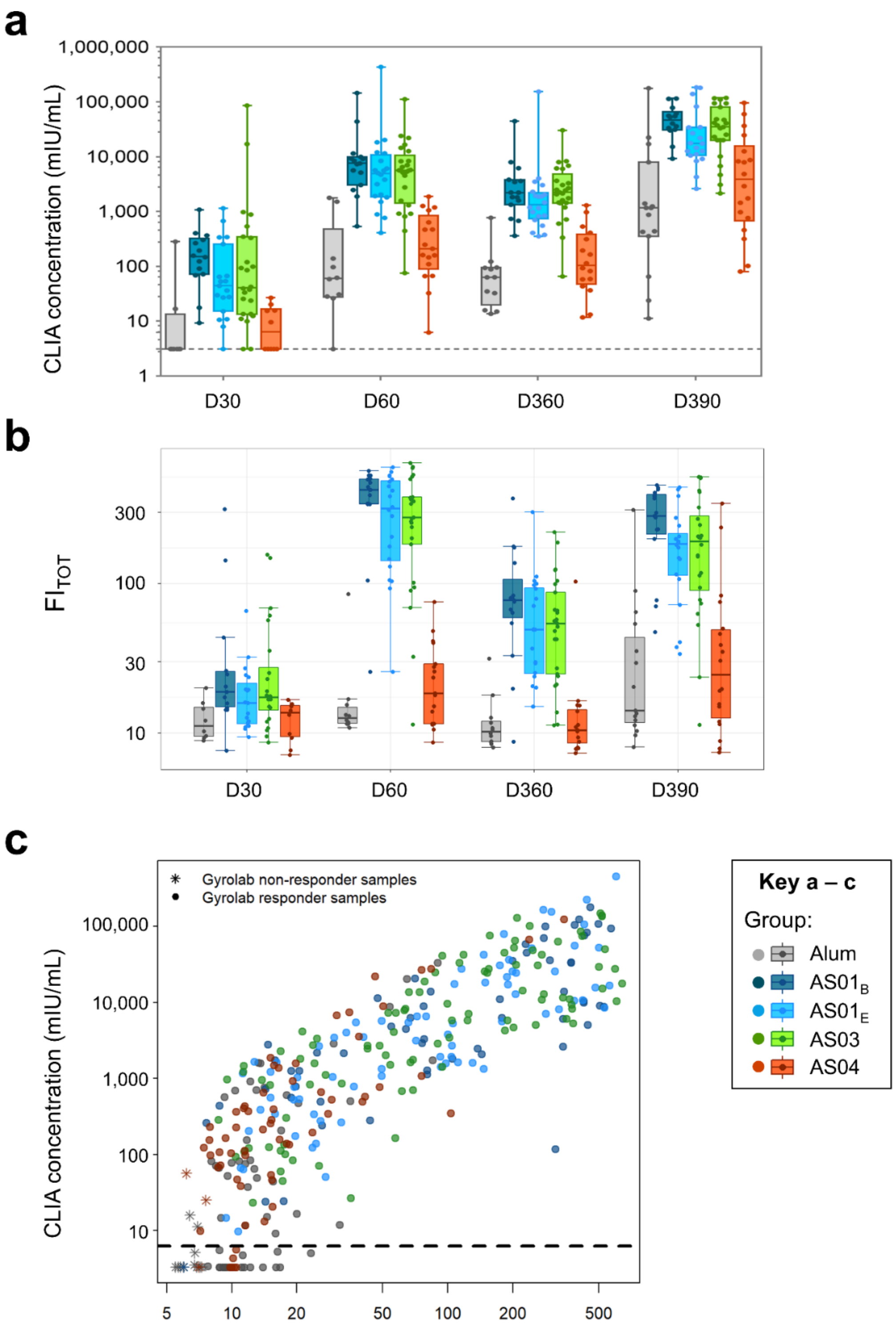
a
CLIA concentration (mIU/mL)
1,000,000
100,000
10,000
1,000
100
10
1
D30
D60
D360
D390
b
FI_{TOT}
300
100
30
10
D30
D60
D360
D390
c
Gyrolab non-responder samples
Gyrolab responder samples
CLIA concentration (mIU/mL)
100,000
10,000
1,000
100
10
5
10
20
50
100
200
500
Key a – c
Group:
Alum
$AS01_B$
$AS01_E$
AS03
AS04

Supplementary Figure 5. Data analysis pipeline. Figure represents the data analysis steps of the deconvolution algorithm applied to estimate the avidity of serum samples. The input was the polyclonal antibody (pAb) profile representing fluorescence intensity (FI) data obtained by Gyrolab immunoassay. Both the pAb profile, and the bootstrap profiles generated from the pAb profile, were used to determine m_1, i.e., the radial position of the FI peak of the first component (Step 1). The 'bias' in the formula represents a constant correction term to minimize the error between expected and real values, equal to -3. The starting point, SP_{MIX}, was measured directly from the pAb profile, and q, a CD-specific variable, was estimated from the bootstrap profiles. The model selection (Step 2) was performed by fitting approximated Landau probability distributions $L(x)$ to the pAb profiles, using the m_1 values determined in Step 1. The selection of a 1-, 2-, or 3-component model was guided by the Bayesian Information Criterion (BIC) score, and pre-specified criteria for the relative FI value (%FI) and the normalization parameter A (see Section 2.2). To determine the avidity parameters of each component (Step 3), non-linear least-square (NLS) regressions were performed on the bootstrap profiles using the selected model (of note, for the current data-set, only 1- or 2-component descriptions could be detected). Per component, a set of A, m, and W values was estimated by averaging the bootstrap results. The graph in Step 3 shows an example of a 2-component regression. Blue and red curves represent the first and second components, respectively, the green curve represents the sum of the two components, and the black symbols and error bars represent the pAb curve with 95% confidence interval.

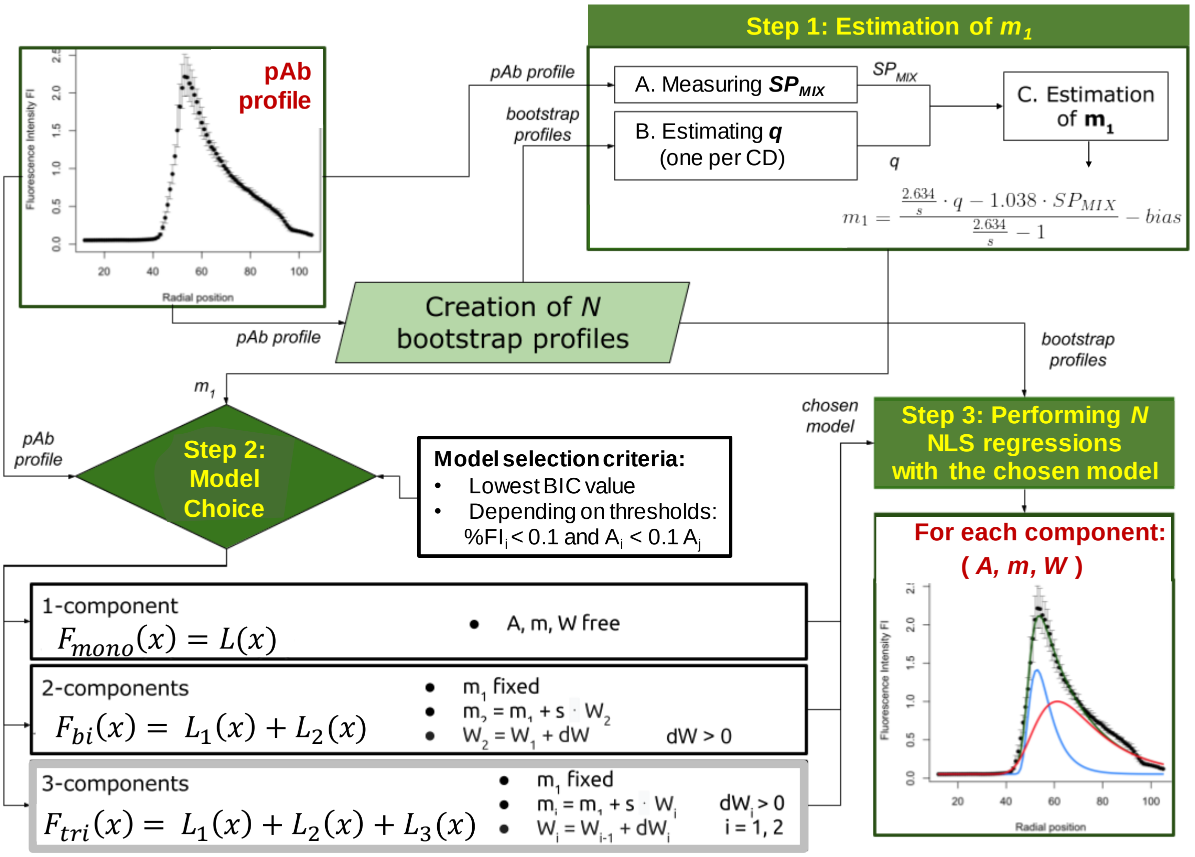
Step 1: Estimation of m1
pAb profile
A. Measuring SPMIX
bootstrap profiles
B. Estimating q
(one per CD)
SPMIX
q
C. Estimation of m1
$m_1 = \frac{\frac{2.634}{s}\cdot q - 1.038\cdot SP_{MIX}}{\frac{2.634}{s} - 1} - bias$
pAb profile
Fluorescence Intensity FI
Radial position
Creation of N bootstrap profiles
pAb profile
bootstrap profiles
m1
chosen model
Step 3: Performing N NLS regressions with the chosen model
pAb profile
Step 2: Model Choice
Model selection criteria:
Lowest BIC value
Depending on thresholds:
%FIi < 0.1 and Ai < 0.1 Aj
For each component:
(A, m, W)
1-component
$F_{mono}(x) = L(x)$
A, m, W free
2-components
$F_{bi}(x) = L_1(x) + L_2(x)$
m1 fixed
m2 = m1 + s · W2
W2 = W1 + dW
dW > 0
3-components
$F_{tri}(x) = L_1(x) + L_2(x) + L_3(x)$
m1 fixed
mi = m1 + s · Wi
Wi = Wi-1 + dWi
dWi > 0
i = 1, 2
