# Supplementary material for: Antibody avidity, persistence, and response to antigen recall: comparison of vaccine adjuvants
Source: NPJ Vaccines. 2021 May 21;6:78. doi: 10.1038/s41541-021-00337-0 (PMC8140094; doi:10.1038/s41541-021-00337-0)
Supplement: Supplementary file 2 — Reporting Summary [file 41541_2021_337_MOESM2_ESM.pdf]

nature research

Corresponding author(s): Wivine Burny

Last updated by author(s): Mar 24, 2021

Reporting Summary

Nature Research wishes to improve the reproducibility of the work that we publish. This form provides structure for consistency and transparency in reporting. For further information on Nature Research policies, see our Editorial Policies and the Editorial Policy Checklist.

Statistics

For all statistical analyses, confirm that the following items are present in the figure legend, table legend, main text, or Methods section.

n/a	Confirmed	
☐	☒	The exact sample size (n) for each experimental group/condition, given as a discrete number and unit of measurement
☐	☒	A statement on whether measurements were taken from distinct samples or whether the same sample was measured repeatedly
☐	☒	The statistical test(s) used AND whether they are one- or two-sided<br>*Only common tests should be described solely by name; describe more complex techniques in the Methods section.*
☒	☐	A description of all covariates tested
☒	☐	A description of any assumptions or corrections, such as tests of normality and adjustment for multiple comparisons
☒	☐	A full description of the statistical parameters including central tendency (e.g. means) or other basic estimates (e.g. regression coefficient) AND variation (e.g. standard deviation) or associated estimates of uncertainty (e.g. confidence intervals)
☒	☐	For null hypothesis testing, the test statistic (e.g. F, t, r) with confidence intervals, effect sizes, degrees of freedom and P value noted<br>*Give P values as exact values whenever suitable.*
☒	☐	For Bayesian analysis, information on the choice of priors and Markov chain Monte Carlo settings
☒	☐	For hierarchical and complex designs, identification of the appropriate level for tests and full reporting of outcomes
☒	☐	Estimates of effect sizes (e.g. Cohen's d, Pearson's r), indicating how they were calculated

Our web collection on statistics for biologists contains articles on many of the points above.

Software and code

Policy information about availability of computer code

Data collection: Full code used for the analyses presented in this manuscript is available upon request to the corresponding author.

Data analysis: Full code used for the analyses presented in this manuscript is available upon request to the corresponding author.

For manuscripts utilizing custom algorithms or software that are central to the research but not yet described in published literature, software must be made available to editors and reviewers. We strongly encourage code deposition in a community repository (e.g. GitHub). See the Nature Research guidelines for submitting code & software for further information.

Data

Policy information about availability of data

All manuscripts must include a data availability statement. This statement should provide the following information, where applicable:

- Accession codes, unique identifiers, or web links for publicly available datasets
- A list of figures that have associated raw data
- A description of any restrictions on data availability

GSK makes available anonymized individual participant data and associated documents from interventional clinical studies which evaluate medicines, upon approval of proposals submitted to www.clinicalstudydatarequest.com. To access data for other types of GSK sponsored research, for study documents without patient-level data and for clinical studies not listed, please submit an enquiry via the website (ClinicalTrials.gov identifier: NCT00805389).

Field-specific reporting

Please select the one below that is the best fit for your research. If you are not sure, read the appropriate sections before making your selection.

☒ Life sciences ☐ Behavioural & social sciences ☐ Ecological, evolutionary & environmental sciences

For a reference copy of the document with all sections, see nature.com/documents/nr-reporting-summary-flat.pdf

Life sciences study design

All studies must disclose on these points even when the disclosure is negative.

Sample size	Participants were healthy HBV-naïve men and women aged 18–45 years, who were randomized (1:1:1:1:1) to receive two doses of 20 µg HBsAg vaccine adjuvanted with AS01B, AS01E, AS03, AS04 or Alum. As pre-specified analyses, persistence and boostability of the adaptive immune response were evaluated in the Booster ATP immunogenicity cohort ('Booster cohort'; N=265), and in a cohort subset (N=99). Antibody avidity (a post-hoc analysis) was evaluated at D30, D60, D360 and D390. Analyses were performed for the Booster cohort subset (N=99), excluding three subjects who were (CLIA) seronegative on all four time-points.
Data exclusions	N/A
Replication	N/A
Randomization	N/A
Blinding	N/A

Reporting for specific materials, systems and methods

We require information from authors about some types of materials, experimental systems and methods used in many studies. Here, indicate whether each material, system or method listed is relevant to your study. If you are not sure if a list item applies to your research, read the appropriate section before selecting a response.

Materials & experimental systems

n/a	Involved in the study	
☐	☒	Antibodies
☒	☐	Eukaryotic cell lines
☒	☐	Palaeontology and archaeology
☒	☐	Animals and other organisms
☐	☒	Human research participants
☐	☒	Clinical data
☒	☐	Dual use research of concern

Methods

n/a	Involved in the study	
☒	☐	ChIP-seq
☐	☒	Flow cytometry
☒	☐	MRI-based neuroimaging

Antibodies

Antibodies used	Described previously (Leroux-Roels, G. et al. Impact of adjuvants on CD4+ T cell and B cell responses to a protein antigen vaccine: Results from a phase II, randomized, multicenter trial. Clin. Immunol 169, 16-27 (2016))
Validation	N/A

Human research participants

Policy information about studies involving human research participants

Population characteristics	Described previously (Leroux-Roels, G. et al. Impact of adjuvants on CD4+ T cell and B cell responses to a protein antigen vaccine: Results from a phase II, randomized, multicenter trial. Clin. Immunol 169, 16-27 (2016))
Recruitment	Described previously (Leroux-Roels, G. et al. Impact of adjuvants on CD4+ T cell and B cell responses to a protein antigen vaccine: Results from a phase II, randomized, multicenter trial. Clin. Immunol 169, 16-27 (2016))
Ethics oversight	Described previously (Leroux-Roels, G. et al. Impact of adjuvants on CD4+ T cell and B cell responses to a protein antigen vaccine: Results from a phase II, randomized, multicenter trial. Clin. Immunol 169, 16-27 (2016))

Note that full information on the approval of the study protocol must also be provided in the manuscript.

Clinical data

Policy information about clinical studies

All manuscripts should comply with the ICMJE guidelines for publication of clinical research and a completed CONSORT checklist must be included with all submissions.

Clinical trial registration: ClinicalTrials.gov identifier: NCT00805389

Study protocol: GSK makes available anonymized individual participant data and associated documents from interventional clinical studies which evaluate medicines, upon approval of proposals submitted to www.clinicalstudydatarequest.com. To access data for other types of GSK sponsored research, for study documents without patient-level data and for clinical studies not listed, please submit an enquiry via the website (ClinicalTrials.gov identifier: NCT00805389).

Data collection: The observer-blind, randomized, controlled trial (ClinicalTrials.gov identifier: NCT00805389) was conducted from December 2008 to July 2011. Last collected data in 2019.

Outcomes: N/A

Flow Cytometry

Plots

Confirm that:

- [ ] The axis labels state the marker and fluorochrome used (e.g. CD4-FITC).
- [ ] The axis scales are clearly visible. Include numbers along axes only for bottom left plot of group (a 'group' is an analysis of identical markers).
- [ ] All plots are contour plots with outliers or pseudocolor plots.
- [x] A numerical value for number of cells or percentage (with statistics) is provided.

Methodology

Sample preparation: Described previously (Leroux-Roels, G. et al. Impact of adjuvants on CD4+ T cell and B cell responses to a protein antigen vaccine: Results from a phase II, randomized, multicenter trial. Clin. Immunol 169, 16-27 (2016))

Instrument: Described previously (Leroux-Roels, G. et al. Impact of adjuvants on CD4+ T cell and B cell responses to a protein antigen vaccine: Results from a phase II, randomized, multicenter trial. Clin. Immunol 169, 16-27 (2016))

Software: Described previously (Leroux-Roels, G. et al. Impact of adjuvants on CD4+ T cell and B cell responses to a protein antigen vaccine: Results from a phase II, randomized, multicenter trial. Clin. Immunol 169, 16-27 (2016))

Cell population abundance: Described previously (Leroux-Roels, G. et al. Impact of adjuvants on CD4+ T cell and B cell responses to a protein antigen vaccine: Results from a phase II, randomized, multicenter trial. Clin. Immunol 169, 16-27 (2016))

Gating strategy: Described previously (Leroux-Roels, G. et al. Impact of adjuvants on CD4+ T cell and B cell responses to a protein antigen vaccine: Results from a phase II, randomized, multicenter trial. Clin. Immunol 169, 16-27 (2016))

- [ ] Tick this box to confirm that a figure exemplifying the gating strategy is provided in the Supplementary Information.
